# Spectrum of Multiple Congenital Anomalies-Hypotonia-Seizures Syndrome 3 (MCAHS3) Due to Phosphatidylinositol Glycan Biosynthesis Class T (PIGT) Gene Mutations: A Narrative Review

**DOI:** 10.7759/cureus.60737

**Published:** 2024-05-21

**Authors:** Ankit Ranjan, Md Shahbaz Alam, Vinod Kumar, Rajesh Kumar, Khalid M Saifullah, Sofia Fakih

**Affiliations:** 1 Department of Neonatology, Rani Hospital, Ranchi, IND

**Keywords:** multiple congenital anomalies-hypotonia-seizures syndrome 3, pigt, pediatric seizure disorder, craniofacial dysmorphism, gpi deficiency, mcahs3

## Abstract

Multiple congenital anomalies-hypotonia-seizures syndrome 3 (MCAHS3) results from mutations in the phosphatidylinositol glycan biosynthesis class T (PIGT) gene leading to defects in glycosylphosphatidylinositol transamidase complex (GPI-TA) synthesis. Glycosylphosphatidylinositol serves as an anchor to more than 150 mammalian proteins for attachment on cell surfaces, enabling specific functional properties. Mutations in the PIGT gene result in disruption of this extremely important post-translational protein modification, yielding dysfunctional proteins leading to MCAHS3.

An exhaustive literature search was conducted across various electronic databases to reveal only 41 reported cases of MCAHS3 worldwide, emphasizing the rarity of this condition. Multiple congenital anomalies-hypotonia-seizures syndrome 3 has been reported as secondary to 18 different known PIGT variants to date, manifesting as a varying spectrum of craniofacial dysmorphism, developmental delay with epilepsy, cardiac and renal malformations, and unique features in biochemical testing and neuroimaging. This review aims to highlight the constellation of clinical symptoms, diagnostic modalities, and management challenges associated with MCAHS3 cases. It would help determine optimal diagnostic and treatment strategies for newly identified cases and facilitate new research on this rare condition.

## Introduction and background

The expression of mammalian eukaryotic proteins on the plasma membrane involves specific anchor molecules. Glycosylphosphatidylinositol (GPI) acts as a lipid anchor for more than 150 such mammalian proteins. The specific anchor maturation involves the assembly of GPI on a phosphatidylinositol lipid structure inside the endoplasmic reticulum, which undergoes a series of enzymatic modifications. The next major modification involves the covalent attachment of the GPI anchor to the carboxyl-terminus of the respective protein moieties. This attachment of GPI to proteins is mediated by a multi-subunit enzymatic complex called glycosylphosphatidylinositol transamidase (GPI-TA). It recognizes and cleaves the carboxy-terminal GPI attachment signal of precursor proteins, followed by the attachment of GPI to the exposed carboxyl-terminus, resulting in the formation of glycosylphosphatidylinositol anchor protein (GPI-AP) [1]. The GPI-AP assembly is transported after final maturation in the Golgi apparatus to the cell surface, playing a crucial role as transcytotic receptors, adhesion molecules, protease inhibitors, complement regulators, and signal transduction pathway co-receptors [2]. With the advent of recent genetic tests such as exome sequencing, a number of diseases have been unraveled secondary to loss of function mutations in genes related to GPI-AP assembly, lipid-protein attachment, and post-translational remodeling. The role of biallelic variants involved in the GPI-AP biosynthetic pathway is increasingly recognized as the cause of disorders like multiple congenital anomalies-hypotonia-seizures syndrome complex (MCAHS), coloboma, congenital heart disease, hyperphosphatasia with mental retardation syndrome, ichthyosiform dermatosis, mental retardation, ear anomalies/epilepsy syndrome, and early infantile epileptic encephalopathy [2,3].

The mammalian GPI-TA structurally exists in a pentameric state comprising five different subunits, namely phosphatidylinositol glycan anchor biosynthesis class K (PIGK), phosphatidylinositol glycan anchor biosynthesis class U (PIGU), phosphatidylinositol glycan anchor biosynthesis class S (PIGS), phosphatidylinositol glycan anchor biosynthesis class T (PIGT), and glycosylphosphatidylinositol anchor attachment 1 (GPAA1). Loss of function mutation in any of the genes encoding these subunits leads to multi-system disorders including developmental delay, hypotonia, craniofacial dysmorphism, psychomotor retardation, complex seizure semiology, cardiovascular, genitourinary, and skeletal system anomalies [4]. These constellations of symptoms have been grouped under the umbrella of a rare syndrome complex called MCAHS. The PIGT subunit plays a critical role in the generation of carbonyl intermediates, facilitating protein attachment. Furthermore, PIGT confers stability to the GPI-TA complex by linking the PIGS subunit to the GPAA1 subunit [5].

Homozygous or compound heterozygous mutations in the PIGT gene on chromosome 20q13 presenting with the above spectrum are classified as MCAHS3. Although the major symptoms of PIGT gene mutations are similar to those of other GPI-AP biosynthetic defects, many peculiar associations have been described with MCAHS3. High expression of the PIGT gene in the central nervous system (CNS) has been postulated as the reason for severe neurological symptoms as compared to other GPI-AP subunit disorders. Hair, skin, and nail anomalies have been unreported in the MCAHS3 subtype, as noted in patients with other MCAHS subtypes [6].

Any cases fitting into the probable spectrum of MCAHS3 can be potentially diagnosed with whole-exome sequencing (WES) to identify the presence of pathogenic PIGT variants, followed by Sanger’s sequencing to confirm the sequence variant. Flow cytometric analysis of granulocytes isolated from whole blood for expression of specific GPI-anchored proteins also aids in diagnosis. Using antibodies directed to CD16, CD55, CD59, CD66b, and fluorochrome-conjugated aerolysin (FLAER); the expression of markers may be evaluated as they specifically bind to GPI anchors by comparing the mean fluorescence intensity between control and cases [6-8]. This review presents a comprehensive analysis of all published cases of MCAHS3 to highlight the PIGT variants reported to date along with the expected constellation of signs and symptoms and diagnostic laboratory and neuroimaging findings. 

## Review

Methods

A systematic search of the literature was conducted on the National Institute of Health’s PubMed, Scopus, Medical Literature Analysis and Retrieval System Online (MEDLINE), Google Scholar, and Web of Science databases. The keywords “multiple congenital anomalies-hypotonia-seizures syndrome” OR “PIGT” OR “MCAHS3” were used. No limitation was used on publication years or study design owing to the rarity of this condition to include all relevant literature available. The systematic search generated 102 articles. Studies published in peer-reviewed journals in the English language were taken into account for further screening. The reference list of the published articles was also screened to improve the sensitivity of the search process. Full texts of all such articles were extracted, followed by the removal of all duplicate items after screening the titles, year of publication, and authors. Two authors independently studied the articles to exclude the non-relevant ones. Any conflicts between the two authors were settled by consensus or, if needed, by consulting a third reviewer. After filtering out duplicate and irrelevant literature, a total of 15 articles were identified, describing only 41 cases across the world (Table 1) with the pathological PIGT mutation [5-19]. Anthropometric data (if available at birth) was plotted on modified Fenton's charts or World Health Organization Multicentre Growth Reference Study (WHOMGRS) charts depending on preterm or term gestation, respectively [20]. Clinical and genetic details, along with important laboratory investigations, were recorded in a pre-designed case record form and transferred to a Microsoft Excel spreadsheet (Microsoft Corp., Redmond, WA) for further analysis. After a thorough evaluation, the results were synthesized and presented narratively.

**Table 1:** Overview of demographic, genetic, clinical, MRI, and laboratory features of published MCAHS3 cases MCAHS3: multiple congenital anomalies-hypotonia-seizures syndrome 3 ; M: male; F: Female; Y: years; MC: myoclonic seizures; GTCS: generalized tonic-clonic seizures; AS: absence seizures; TS: tonic seizures; GDD: global developmental delay; PDA: patent ductus arteriosus; ASD: atrial septal defect; ALP: alkaline phosphatase; IgA: immunoglobulin A; IgM: immunoglobulin M

Characteristics	Kvarnung et al., 2013 [10]	Nakashima et al., 2014 [16]	Lam et al., 2015 [7]	Skauli et al., 2016 [14]	Pagnamenta et al., 2017 [13]	Kohashi et al., 2017 [11]	Yang et al., 2018 [6]	Bayat et al., 2019 and 2021 [5,8]	Jezela-Stanek et al., 2020 [17]	Mason et al., 2019 [12]	Jiao et al., 2020 [9]	Hur et al., 2021 [19]	Sai Chander et al., 2021 [15]	Ranjan et al., 2023 [18]
	4 cases	1 case	2 cases	2 cases	2 cases	1 case	1 case	15 cases	7 cases	1 case	2 cases	1 case	1 case	1 case
Ethnicity	Turkish	Japanese	Caucasian mother	African	Caucasian	Japanese	Chinese	Polish( 4)	Polish	Greek	Chinese	Korean	Indian	Indian
								Danish (2)						
								Somalian (2)						
								Pakistani (1)						
								Bangladeshi (2)						
								Caucasian (2)						
			Afro-American father		Afghanistan			Not specified(2)						
Age at diagnosis/Sex	1-3Y/ F(4/4)	12Y/F	7Y/F	9Y/M	Not specified/F	11 months/M	11 months/ M	4-9Y/All F	6 months to 16Y/M(4), F(3)	18 months/M	13Y/M	35 months/F	11 months/F	11 months/M
			6Y/M	7Y/M	Not specified/ M			11 months/M and 7Y/F			1Y/M			
								22 months and 11Y/M(2)						
								2Y/F						
								4 months and 6 months/F(2)						
								2Y and 7.5 Y/F(2)						
								Not specified/F and 26 months/M						
PIGT variant	c.547A>C; c.547A>C	c.250G>T; c.1342C>T	c.918dupC; c.1342C>T	c.1079G>T; c.1079G>T	Pt 1: c.1582G>A; c.1730dupC	c.250G>T; c.1096G>T	c.550G>A; c.550G>A	Polish- 4 patients c.1582G>A; c.1582G>A (2)	c.1582G>A; c.1582G>A (2/7) c.1582G>A; c.1520G>A (1/7)	c.547A>C; c.494-2A>G	c.469T>G; c.1120A>G	c.250G>T; c.1582G>A	c.1342C>T; c.1342C>T	c.709G>C; c.709G>C
								c.494-2A>G; c.1582G>A (1)						
								c.1730dupC; c.1582G>A(1)	c.1582G>A; c.494-2A>G (2/7)					
								Danish- c.1472T>A; c.1484+2T>A (2)						
								Somalian and other not specified- c.1079G>T; c.1079G>T (4)	c.1582G>A; c.1730dupC (1/7)		c.514C>T; c.98delA			
								Pakistani- c.550G>A; c.550G>A						
					Pt 2: c.709G>C; c.709G>C			Bangladeshi- c.709G>C; c.709G>C (2)	c.1582G>A; c.1096G>A (1/7)					
								Caucasian- c.1582G>A; c1582G>A (2)						
Gestational age at birth (weeks)	37-40	40	31 (both)	40 (both)	Not specified	Not specified	40	29 - 42	37-41	38	37	39	38	38
Birth weight(centile)	69^th^-99^th^	50^th^-90^th^	10^th^-90^th^	50^th^	Not specified	Not specified	50^th^	<3^rd^ – 99^th^	20^th^ – 97^th^	25^th^	Not specified	Not specified	36th	21^st^
HC at birth (centile)	84^th^-99^th^	50^th^-90^th^	<10^th^-75^th^	10^th^ - >97^th^	Not specified	Not specified	50^th^	<3^rd ^– >95^th^	3^rd^ – 90^th^	25^th^	Not specified	Not specified	Not specified	80^th^
Length at birth (centile)	93^th^-99^th^	50^th^	10^th^-90^th^	50^th^	Not specified	Not specified	50^th^	75^th^ – 97^th^	70^th^- 99th	<3^rd^	Not specified	Not specified	Not specified	60^th^
Dysmorphism														
Skull	Brachycephaly (4/4)	Not specified	Brachycephaly (2/2)	Not specified	Not specified	Not specified	Not specified	Brachycephaly (5/15)\	Not specified (7/7)	Plagiocephaly	Not specified (2/2)	Frontal bossing	Not specified	Yes
								Normal (2/15)						
								Not specified (4/15)						
High forehead	Yes (4/4)	Not specified	Yes (2/2)	Yes (2/2)	Yes (1/2)	Not specified	Yes	Yes (12/15)	Not specified (7/7)	Yes	Not specified (2/2)	Not specified	Yes	Yes
								Not specified (3/15)						
Bitemporal narrowing	Yes (4/4)	Not specified	Yes (2/2)	Yes (2/2)	Yes (1/2)	Not specified	Yes	Yes (6/15)	Not specified (7/7)	Yes	Not specified (2/2)	Yes	No	Yes
								Not specified (3/15)						
Telecanthus	Not specified	Not specified	Not specified	Not specified	Yes (1/2)	Yes	Not specified	Yes (8/15)	Not specified (7/7)	Not specified	Not specified (2/2)	Not specified	No	No
								Not specified (4/15)						
Eyebrows	Arched (3/4)	Not specified	Arched (2/2)	Arched (1/2)	Straight (1/2)	Arched	Not specified	Straight (5/15)	Not specified (7/7)	Not specified	Not specified (2/2)	Not specified	No	Arched
								Arched (2/15)						
								Not specified (4/15)						
Palpebral fissures	Straight (4/4)	Upslanting	Upslanting (2/2)	Straight (2/2)	Straight (1/2)	Not specified	Straight	Straight (5/15)	Not specified (7/7)	Not specified	Not specified (2/2)	Not specified	No	Straight
								Upslanting (3/15)						
								Downslanting (1/15)						
								Not specified (3/15)						
Short, anteverted nose	Yes (4/4)	Yes	Yes (2/2)	Yes (2/2)	Yes (1/2)	Yes	Yes	Yes (6/15)	Not specified (7/7)	Yes	Not specified (2/2)	Yes	Not specified	Yes
								Not specified (5/15)						
Depressed nasal bridge	Yes (4/4)	Yes	Yes (2/2)	Yes (2/2)	Not specified	Yes	Yes	Yes (8/15)	Not specified (7/7)	No	Yes (1/2)	Not specified	Yes	Yes
								Not specified (4/15)						
Philtrum	Long (4/4)	Not specified	Long (2/2)	Short (2/2)	Short (1/2)	Long	Long	Long (4/15)	Not specified (7/7)	Long	Not specified (2/2)	Short	Long	Long
								Short (1/15)						
								Not specified (3/15)						
Tented upper lip	Yes (4/4)	Not specified	Yes (2/2)	Not specified	Not specified	Yes	Yes	Yes (9/15)	Not specified (7/7)	Yes	Not specified (2/2)	Yes	No	Yes
								Not specified (4/15)						
High arched palate	Not specified	Yes	Yes (2/2)	Yes (2/2)	Not specified	Yes	Yes	Yes (7/15)	Not specified (7/7)	Yes	Yes (1/2)	Not specified	Yes	Yes
								Not specified (6/15)						
Teeth abnormalities	Yes (4/4)	No	Not specified	Not specified	Not specified	Not specified	Yes	Yes (5/15)	Not specified (7/7)	Not specified	Not specified (2/2)	No	Not specified	Delayed dentition
								Not specified (6/15)						
Micrognathia	Yes (4/4)	Yes	No	No	No	Yes	Not specified	Yes (2/15)	Not specified (7/7)	Not specified	Not specified (2/2)	Not specified	No	No
								Not specified (3/15)						
Low set ears	Not specified	Yes	Large ears (2/2)	Yes (1/2)	Yes (1/2)	Not specified	Yes	Yes (3/15)	Not specified (7/7)	Yes	Yes (1/2)	Yes	Yes	Yes
								Not specified (3/15)						
Hypotrichosis	Yes (4/4)	Not specified	Yes (2/2)	Yes (2/2)	Not specified	Not specified	Not specified	Yes (8/15)	Not specified (7/7)	Not specified	Not specified (2/2)	Not specified	Not specified	No
								Not specified (7/15)						
Hypotonia	Yes (4/4)	Yes	Yes (2/2)	Yes (2/2)	Yes (2/2)	Yes	Yes	Yes (15/15)	Yes (7/7)	Yes	Yes (1/2)	Yes	Yes	Yes
								Brisk reflexes (2/15)						
Seizure onset	12-18 months	4 months	5 months each	12 months (both)	Pt 1: 12 months	2 months	1 month	1^st^ day (3)- Danish (2) and Bangladeshi (1)	6-12 month	5 month	3yrs (1/2)	8 months	11 month	Day 1
					Pt 2: Neonatal			2^nd^ week– Bangladeshi (1)			4 month (1/2)			
Seizure semiology	MCS, GTCS, AS	MCS, GTCS, Apneic spells	MCS, GTCS, TS	MCS, TS, GTCS	GTCS, MCS	MCS, TS, apneic spells	MCS, febrile seizures	MCS, GTCS, TS, apneic spells, atonic, febrile seizures	Fever-associated seizures (7/7) mostly GTCS, MCS	FS, GTCS	FS, MCS	FS, GTCS	GTCS, choreoathetoid movements	MCS, GTCS, TS
EEG findings	Multifocal epileptiform abnormalities (3/4)	Multifocal epileptiform abnormalities	Multifocal epileptiform abnormalities (2/2)	Multifocal epileptiform abnormalities (2/2)	Multifocal epileptiform abnormalities (2/2)	Multifocal epileptiform abnormalities	Absent slow-wave sleep (SWS), background slowing	Multifocal epileptiform abnormalities (1)	Spike-polyspike wave complex (1) Multifocal epileptiform abnormalities (2)	Not specified	Multifocal epileptiform abnormalities (1); Left anterior and middle temporal discharge (1)	Diffuse slow-wave background rhythms	Diffuse background slowing	Not available
								Not specified (2)						
								Background slowing (7)						
								Burst suppression (5)						
Epileptic encephalopathy	Yes (4/4)	Yes	Yes (2/2)	Yes (2/2)	Yes (Case 2)	Yes	GDD with epilepsy	Yes (9/15)	None (0/7)	GDD with epilepsy	Not specified	No	No	Yes
								GDD with epilepsy (5/15)						
Seizure outcome	Intractable	Intractable	Intractable	Intractable	Case 1: Favorable	Intractable	Intractable	Intractable (9/15)	Favorable (6/7)	Intractable	Intractable (2/2)	Favorable	Favorable	Intractable
					Case 2: Intractable			Favorable (6/15)						
Intellectual disability	Profound (4/4)	Profound	Profound (2/2)	Profound (2/2)	Profound (2/2)	Profound	Profound	Profound (9/15)	Moderate (7/7)	Profound	Not specified	Not specified	Not specified	Not specified
								Severe (4/15)						
								Not specified(2/15)						
MRI brain: cerebral and cerebellar atrophy	Yes (3/4)	Yes	Yes (2/2)	Yes (2/2)	Isolated cerebellar atrophy (case 1)	Isolated cerebellar atrophy	Yes	Yes (6/15)	Cerebellar atrophy (5/7)	No	Normal (1)	Cerebellar atrophy	Isolated cerebral atrophy	Not available
								Isolated cerebellar atrophy (1/15)			Enlarged subarachnoid space (1)			
OPHTHALMOLOGICAL FINDINGS														
Strabismus, nystagmus	Yes (4/4)	Yes	Yes (2/2)	Yes (2/2)	Yes (Case 2)	Not specified	Yes	Yes (8/15)	Yes (7/7)	Yes	Not specified (2/2)	Not specified	Yes	Yes
								Not specified (3/15)						
Cortical visual impairment	Yes (4/4)	Yes	Yes (2/2)	Yes (2/2)	Not specified	Not specified	Not specified	Yes (6/15)	Not specified	Yes	Not specified (2/2)	Not specified	No	Could not be tested
								Not specified (4/15)						
Other findings	Downward gaze paresis (1/4)	None	Astigmatism (1/2)	Astigmatism (2/2)	Oculomotor apraxia (Pt 1);	Not specified	Not specified	Hyperopia (4/15)	None	Hypermetropia	Not specified (2/2)	None	None	None
					Optic atrophy (Pt 2)			Myopia (1/15)						
AUDITORY DEFICIT	No (0/4)	Not specified	Yes (2/2)	Not specified	Not specified	Not specified	No	No (9/15)	Not specified (7/7)	No	Not specified (2/2)	Not specified	No	No
								Not specified (4/15)						
CONGENITAL ANOMALIES														
Cardiovascular	PDA (1/4)	PDA	ASD requiring device closure (1/2)	None	Not specified	None	None	PDA (1/15)	Not specified (7/7)	Dextroposed heart	Not specified (2/2)	None	None	Small ASD (3 mm) with left to right shunt
	Restrictive cardiomyopathy (1/4)							Not specified (2/15)						
Genitourinary	Nephrocalcinosis (4/4)	Ureteral dilatation	None	None	Nephrolithiasis (Pt 1)	Not specified	Not specified	Nephrocalcinosis (1/15)	Not specified (7/7)	Ureteric dilatation, renal cysts with dysplasia, grade V VUR	Not specified (2/2)	None	None	None
	Renal cysts (1/4)							Ureteral dilatation(1/15)						
	Ureteral dilatation(3/4)							Not specified (2/15)						
Musculoskeletal	Slender long bones (4/4)	Scoliosis	Slender long bones with scoliosis (2/2)	None	Not specified	Not specified	Not specified	Slender long bones (3/15)	Not specified (7/7)	Pectus excavatum, clinodactyly, syndactyly, short limbs, talipes equinovarus	Not specified (2/2)	None	None	Congenital fractures, clinodactyly, bilateral digital contractures
								Scoliosis (1/15)						
								Craniosynostosis (1/15)						
								Pectus excavatum (1/15)						
	Scoliosis with craniosynostosis (2/4)							Joint hypermobility (5/15)						
	Pectus excavatum (1/4)							Congenital fractures (1) (Bangladeshi origin)						
								Not specified (2/15)						
Respiratory	Atypical lung lobulation (1/4)	Not specified	Mixed apnea (1/2)	None	Not specified	Central apnea	Recurrent respiratory infection	Central sleep apnea (4/15)	Not specified (7/7)	Recurrent aspiration pneumonia	Not specified (2/2)	None	None	Recurrent respiratory infection
								Obstructive apnea (2/15)						
								Not specified (3/15)						
LAB														
ALP levels	Low (4/4)	Low	Normal (2/2)	Normal (2/2)	Pt 1: Normal; Pt 2: Low	Low	Normal	Normal (12/15)	Not specified (7/7)	Normal	Normal (1/2) Not specified (1/2)	Low	Normal	Low
								Not specified (3/15)						
Miscellaneous	None	None	IgA and IgM deficiency, hypertriglyceridemia	None	None	None	None	IgA and IgM deficiency (2/15)	Hypoglycemia (2/7) (In two heterozygotic patients)	Undescended testis, bilateral inguinal hernia	Café-au-lait spot (1/2)	None		Multiple episodes of ventilator-associated pneumonia
Plasma Calcium	High	Normal	Normal	Not specified	Normal	Normal	Normal	Normal (4/13) and Not specified (9/13)	Not specified (7/7)	Normal	Not specified (2/2)	Not specified	Not specified	Normal
Plasma phosphate	Normal	Not specified	Normal	Not specified	Normal	Normal	Normal	Normal (10/13)/ Not specified (3/13)	Not specified (7/7)	Normal	Not specified (2/2)	Not specified	Not specified	Normal

Statistical analyses

Data were presented as mean ± standard deviation for continuous variables and medians with interquartile ranges for skewed distributions. Categorical variables were summarized using frequencies and percentages. IBM SPSS Statistics Software for Windows, version 29 (IBM Corp., Armonk, NY) was used for statistical analysis.

Results

The 41 cases of MCAHS3 obtained after a thorough literature search were analyzed in detail. Tables 1-2 show the demographic characteristics, the spectrum of PIGT variants, dysmorphic features, details of neurological abnormalities, the gamut of congenital anomalies, and relevant laboratory parameters in all 41 cases with pathological PIGT mutations, along with their relative prevalence.

**Table 2:** Relative prevalence of demographic variables and clinical parameters in cases with pathological PIGT mutations n: number of cases in which that particular variable was reported

Serial number	Clinical variables	Frequency (Percentage)
1.	Sex distribution (male/female) (n=41)	18 (44%)/ 23 (56%)
2.	Gestational age at birth (n=38)	
	Term (≥37 weeks)	35 (92.1 %)
	Preterm (<37 weeks)	3 (7.9%)
3.	Consanguinity (n=34)	7 (20.5%)
4.	Age at onset of seizures (n=41)	
	Neonatal (0-28 days)	6 (14.6%)
	Infantile (>28 days till <1 year)	20 (48.8%)
	More than 1 year	15 (36.6%)
5.	Anthropometry	
	Head circumference (n=29)	2(6.8%)/12 (41.3%)
	<3^rd^ centile/>90^th^ centile	
	Weight (n=35)	2(5.7%)/12(34.3%)
	<3^rd^ centile/>90^th^ centile	
	Length (n= 27)	1(3.7%)/14(51.8%)
	<3^rd^ centile/>90^th^ centile	
6.	Hypotonia (n=41)	41 (100%)
7.	Brisk reflexes (n=41)	4 (9.7%)
8.	Epileptic encephalopathy (n=40)	22 (55%)
9.	Intractable seizures (n=41)	26 (63.4%)
10.	Severe to profound intellectual disability (n=34)	27 (79.4%)
11.	Electroencephalography findings (n=37)	
	Multifocal epileptiform discharges	15 (40.5%)
	Burst suppression	5 (13.5%)
12.	Magnetic resonance imaging of brain findings (n=40)	
	Cerebral and cerebellar atrophy	18(45%)
	Predominant cerebellar atrophy	4 (10%)
13.	Strabismus/nystagmus (n=34)	30 (88.2%)
14.	Cortical visual impairment (n=22)	16 (72.2%)
14.	Auditory deficit (n=21)	2 (9.5%)
15.	Low alkaline phosphatase levels (n=30)	9 (30%)

The sex distribution assessment showed a female preponderance, with the majority delivering at term gestation. Consanguinity was present only in seven cases. A trend of weight, head circumference, and length at birth of more than 90^th^ centile was noted in the majority of cases. Different forms of craniofacial dysmorphism were reported, but incomplete descriptions were not included in the analysis. Dysmorphic spectrum included brachycephaly (12 out of 15 cases that reported the presence or absence of brachycephaly), high forehead (25/29 cases), bitemporal narrowing (19/29 cases), arched eyebrows (10/27 cases), slanting palpebral fissures (7/29 cases), short and anteverted nose (21/31 cases), depressed nasal bridge (22/31 cases), long philtrum (15/30 cases), tented upper lip (20/26 cases), high arched palate (18/21 cases), teeth abnormalities (11/17 cases), micrognathia (8/23 cases), low set ears (12/26 cases) and hypotrichosis (16/17 cases). Seizure onset was observed in infantile (age >28 days until <12 months) and pediatric (age >12 months) groups in 20 cases and 15 cases, respectively.

Neonatal onset was relatively rare, seen only in six cases, and all were associated with intractable seizures. Neonatal and infantile-onset groups (birth till <12 months) and pediatric-onset (>12 months) groups were subsequently compared with each other to study the impact of gender distribution on the age of seizure onset. All the cases had hypotonia with a diminished deep tendon reflex. However, four cases did report a brisk reflex [5, 14]. More than half of the cases had a severe presentation with epileptic encephalopathy. Intractable seizures were noted in 26 cases. All neonatal-onset cases of MCAHS3 had intractable seizures. Severe to profound intellectual disability was strikingly prevalent. The most common electroencephalography (EEG) finding consisted of multifocal epileptiform discharges, while a few instances of burst suppression patterns and generalized background slowing were also detected. Magnetic resonance imaging of the brain revealed cerebral and cerebellar atrophy as prominent findings, followed by isolated cerebellar atrophy in a few cases. Strabismus, or nystagmus, and cortical visual impairment were identified in a significant proportion of cases. However, auditory deficits were rare, with only two cases reporting the same out of 21 for which an auditory screening was documented, though the nature of hearing loss and investigations undertaken for the same were not specified. Low levels of alkaline phosphatase (ALP) were recorded in nine out of 30 cases tested for the enzyme [10,11,13,16,18,19].

Table 3 illustrates the prevalence of individual congenital anomalies grouped under various organ systems reported to be affected by MCAHS3. Patent ductus arteriosus (PDA) was noted in three cases, although hemodynamic significance was not specified in any of them. Two cases had an ostium-secundum atrial septal defect (OS-ASD), one of which required device closure. An isolated case reported restrictive cardiomyopathy [10]. Genitourinary anomalies consisted of ureteral dilatation, nephrocalcinosis, and renal cysts. Musculoskeletal anomalies in the form of slender, long bones and scoliosis were described. Only two cases to date have reported congenital fractures [5,18]. Central or mixed apnea comprised the predominant respiratory problem, with a single case describing atypical lung lobulations [10]. 

**Table 3:** Systemic anomalies reported with pathological PIGT mutations n: number of cases in which that particular variable was reported

Serial number	Organ system	Frequency (percentage)
1.	Cardiovascular system (n=27)	
	Patent ductus arteriosus	3 (11.1%)
	Atrial septal defect	2 (7.4%)
	Restrictive cardiomyopathy	1 (3.7%)
	Normal	21 (77.8%)
2.	Genitourinary system (n=27)	
	Renal cysts	2 (7.4%)
	Ureteral dilatation	6 (22.2%)
	Nephrocalcinosis	6 (22.2%)
	Normal	13 (48.2%)
3.	Musculoskeletal system (n=27)	
	Scoliosis	6 (22.2%)
	Slender long bones	9 (33.3%)
	Congenital fractures	2 (7.4%)
	Normal	10 (37.1%)
4.	Respiratory system (n=25)	
	Structural lung malformation	1 (4.0%)
	Central/mixed apnea	8 (32%)
	Normal	16 (64%)

Discussion

Mabry et al. (1970) described the first case involving a biosynthetic defect in GPI-AP with four siblings presenting with seizures, mental retardation, and increased ALP levels [21]. Since then, several subtypes of MCAHS have been identified due to mutations in the PIGN, PIGA, PIGT, and PIGQ genes, classified as MCAHS 1, 2, 3, and 4, respectively. We described the spectrum of manifestations specific to PIGT mutations leading to MCAHS3.

The female preponderance in the published cases should be extrapolated with caution, as the number of cases is very small. The first ever PIGT mutation was reported by Kvarnung et al. in 2013 (PIGT variant c.547A>C, p.Thr183Pro) in a consanguineous Turkish family of four patients presenting with craniofacial dysmorphism, hypotonia, generalized osteopenia, renal cysts, and restrictive cardiomyopathy [10]. Since then, 41 cases of MCAHS3 have been reported across the world, with variable genotypic variants. The largest case series of MCAHS3 patients was reported by Bayat et al. with 15 novel patients. A total of 18 different homozygous and compound heterozygous pathogenic variants were described without any clustering, rendering it difficult to establish a genotype-phenotype correlation. Cases with the missense c.1582G>A variant (p.Val528Met) variant seen only in Caucasians had a milder phenotype with medically controlled epilepsy [5,8]. Similar findings of a milder phenotype in the form of well-controlled epilepsy were noted in a case series of seven Polish patients. All the cases had the same Val528Met variant [17]. A recent case report from India described neonatal onset severe phenotypic presentation secondary to the missense pathogenic PIGT variant c.709G>C (p.Glu237Gln) [18]. Only three such cases have been reported of this rare PIGT variant to date. Pagnamenta et al. first reported this variant in an Afghanistani boy in a homozygous state [13]. Subsequently, Bayat et al. described the same variant in female siblings of Bangladesh, pointing to an Asian origin. Neonatal-onset epileptic encephalopathy was the primary symptom in all four cases with this variant. The age of onset of neurological symptoms was different from the infantile-onset seizures often provoked by febrile episodes noted in previously reported cases [5,7,10,14]. A striking finding of congenital fracture was noted with the c.709G>C (p.Glu237Gln) PIGT variant. This finding was first reported by Bayat et al. in one of the Bangladeshi siblings, as described previously. The same variant presenting phenotypically with congenital fractures was also recently described in a neonatal case from India by Ranjan et al. [5,18]. This points to a severe phenotypic correlation with this PIGT variant, as the combination of congenital fractures and neonatal onset CNS manifestations has not been described with any other variant.

In most cases, seizures were classified as generalized tonic-clonic, atonic, myoclonic, or dyscognitive in the pediatric age group, while neonates presented with multifocal, clonic, and myoclonic semiologies. A few cases of epileptic apnea have also been described [8,11,16]. Initial anti-epileptic regimens included phenobarbitone, valproate, phenytoin, levetiracetam, carbamazepine, and clonazepam. The majority of cases were uncontrolled and required the use of a newer generation of anti-epileptics with non-conventional mechanisms of action. These included topiramate, zonisamide, large dosages of phenobarbitone, and the ketogenic diet, albeit with mixed outcomes. Treatment with pyridoxine and pyridoxal phosphate was ineffective. A single case from India did report successful control with add-on pyridoxine therapy [18]. In the few cases of severe epileptic encephalopathy, miscellaneous, rarely used drugs such as potassium bromide and acetazolamide were found to be efficacious [11].

Analyzing the brain MRIs revealed atrophic changes in the cerebrum, cerebellum, and brainstem as a prominent finding, with white matter immaturity and abnormal corpus callosum noted in a few cases [5]. Lam et al. concluded that cerebellar atrophy starts earlier and proceeds rapidly when compared to other parts of the brain [7]. On analyzing the age of onset of the MRI findings, it is evident that cerebellar atrophy is expected at an age of around four years. Thus, there appears to be a fraction of cases in which the finding of cerebellar atrophy may have been missed when an MRI was performed at an early age.

Organ system involvement in MCAHS has been variable, with reports of skeletal, genitourinary, cardiovascular, and respiratory system anomalies. No critical cardiac or renal malformations were noted in the PIGT variant c.709G>C (p.Glu237Gln) with severe neonatal-onset encephalopathy [5,13,18].

On review of laboratory parameters, nine cases manifested low levels of ALP. A plausible hypothesis involves ALPL protein dysfunction due to reduced GPI anchoring, leading to hypophosphatasia and generalized osteopenia. However, a major proportion of patients demonstrated normal ALP levels, underscoring the need for further insight and research in this domain [10,11,13,16,18,19]. 

However, there are limitations to our review. All the studies available for the narrative review were case reports and small case series, as the condition is rare. Many of the cases had missing descriptions of dysmorphic traits, organ involvement, neuroimaging, and systemic involvement, which could have improved the sample number for analyzing that particular characteristic. Since the disease is rare, the data analyzed in this review could be prone to data collection and reporting bias, as the case reports and series were reported from varied healthcare settings and caregivers across the globe. 

## Conclusions

Appropriate sensitization of medical personnel and a high index of suspicion are needed to diagnose and report this condition. Prompt identification would increase the pool of cases and help in the evolution of knowledge regarding clinical, laboratory, and causative PIGT variants. This review expands the range of information regarding PIGT variants, providing exhaustive data on the phenotypic and genotypic variations known to date. The genotype-phenotype correlation is still evolving owing to the wide variability in presentation and the limited number of diagnosed cases. It also highlights the need to elaborately investigate dysmorphic hypotonic newborns along with the described neurological symptoms to pinpoint this potentially diagnosable entity. The combination of automated facial analysis for dysmorphology assessment, deep phenotyping, and NGS would be ideal for correct molecular diagnosis.

Though there aren’t any approved genetic therapies for GPI-AP defects yet, the progressive nature of MCAHS3 advocates for trials of therapies that would slow or stall the neurologic deterioration. Neurological dysfunction as the core feature associated with this disorder indicates a need for selecting a vector capable of crossing the blood-brain barrier for precise delivery of gene therapy. High-quality animal studies to test for a competent vector, an appropriate dose of gene therapy, tissue tropism, and optimum delivery of the corresponding gene could pave the way for further research on human tissue. Alternative therapies in the form of gene editing or modulation of any potential regulatory regions in the PIGT gene could also be explored to improve gene expression.

## References

[1] Kinoshita T (2016). Glycosylphosphatidylinositol (GPI) anchors: biochemistry and cell biology: introduction to a thematic review series. J Lipid Res.

[2] Wu T, Yin F, Guang S, He F, Yang L, Peng J (2020). The glycosylphosphatidylinositol biosynthesis pathway in human diseases. Orphanet J Rare Dis.

[3] Ng BG, Freeze HH (2015). Human genetic disorders involving glycosylphosphatidylinositol (GPI) anchors and glycosphingolipids (GSL). J Inherit Metab Dis.

[4] Liu SS, Jin F, Liu YS (2021). Functional analysis of the GPI transamidase complex by screening for amino acid mutations in each subunit. Molecules.

[5] Bayat A, Knaus A, Juul AW (2019). PIGT-CDG, a disorder of the glycosylphosphatidylinositol anchor: description of 13 novel patients and expansion of the clinical characteristics. Genet Med.

[6] Yang L, Peng J, Yin XM (2018). Homozygous PIGT mutation lead to multiple congenital anomalies-hypotonia seizures syndrome 3. Front Genet.

[7] Lam C, Golas GA, Davids M (2015). Expanding the clinical and molecular characteristics of PIGT-CDG, a disorder of glycosylphosphatidylinositol anchors. Mol Genet Metab.

[8] Bayat A, Pendziwiat M, Obersztyn E (2021). Deep-phenotyping the less severe spectrum of PIGT deficiency and linking the gene to myoclonic atonic seizures. Front Genet.

[9] Jiao X, Xue J, Gong P (2020). Analyzing clinical and genetic characteristics of a cohort with multiple congenital anomalies-hypotonia-seizures syndrome (MCAHS). Orphanet J Rare Dis.

[10] Kvarnung M, Nilsson D, Lindstrand A (2013). A novel intellectual disability syndrome caused by GPI anchor deficiency due to homozygous mutations in PIGT. J Med Genet.

[11] Kohashi K, Ishiyama A, Yuasa S (2018). Epileptic apnea in a patient with inherited glycosylphosphatidylinositol anchor deficiency and PIGT mutations. Brain Dev.

[12] Mason S, Castilla-Vallmanya L, James C (2019). Case report of a child bearing a novel deleterious splicing variant in PIGT. Medicine (Baltimore).

[13] Pagnamenta AT, Murakami Y, Taylor JM (2017). Analysis of exome data for 4293 trios suggests GPI-anchor biogenesis defects are a rare cause of developmental disorders. Eur J Hum Genet.

[14] Skauli N, Wallace S, Chiang SC (2016). Novel PIGT variant in two brothers: expansion of the multiple congenital anomalies-hypotonia seizures syndrome 3 phenotype. Genes (Basel).

[15] Sai Chandar D, Krishna Chaithanya B, Prashanthi M (2021). Homozygous phosphatidylinositol glycan class T mutation in an Indian girl with multiple congenital anomalies-hypotonia-seizures syndrome 3. Cureus.

[16] Nakashima M, Kashii H, Murakami Y (2014). Novel compound heterozygous PIGT mutations caused multiple congenital anomalies-hypotonia-seizures syndrome 3. Neurogenetics.

[17] Jezela-Stanek A, Szczepanik E, Mierzewska H (2020). Evidence of the milder phenotypic spectrum of c.1582G>A PIGT variant: delineation based on seven novel Polish patients. Clin Genet.

[18] Ranjan A, Alam MS, Kumar V, Samanta S, Kumar R, Saifullah KM (2023). Multiple congenital anomalies-hypotonia-seizures syndrome 3 secondary to phosphatidylinositol glycan class T mutation: a neonatal case report. Int J Contemp Pediatr.

[19] Hur YJ, Lee BL, Chung WY, Yu S, Jun KR, Oh SH (2021). Compound heterozygous PIGT mutations in multiple congenital anomalies-hypotonia-seizures syndrome: first case in Korea and characterization by persistent hypophosphatasia. Ann Clin Lab Sci.

[20] Shrestha S, Thakur A, Goyal S, Garg P, Kler N (2016). Growth charts in neonates. Curr Med Res Prac.

[21] Mabry CC, Bautista A, Kirk RF, Dubilier LD, Braunstein H, Koepke JA (1970). Familial hyperphosphatase with mental retardation, seizures, and neurologic deficits. J Pediatr.

